# Oral Health Status (DMFT Index) and Hygiene Practices Among Dental Students in Bulgaria: A Pilot Study

**DOI:** 10.3390/dj14030140

**Published:** 2026-03-03

**Authors:** Boryana Levterova, Zlatina Tomova, Desislav Tomov, Yordanka Uzunova

**Affiliations:** 1 Department of Health Management and Healthcare Economics, Faculty of Public Health, Medical University of Plovdiv, 15A Vassil Aprilov Blvd, 4000 Plovdiv, Bulgaria; 2 Department of Prosthetic Dentistry, Faculty of Dental Medicine, Research Institute at Medical University of Plovdiv, Medical University of Plovdiv, 15A Vassil Aprilov Blvd, 4000 Plovdiv, Bulgaria; zlatina.tomova@mu-plovdiv.bg; 3 Research Institute, Medical University of Plovdiv, 15A Vassil Aprilov Blvd, 4000 Plovdiv, Bulgaria; desislav.tomov@mu-plovdiv.bg; 4 Department of Bioorganic Chemistry, Faculty of Pharmacy, Research Institute at Medical University of Plovdiv, Medical University of Plovdiv, 15A Vassil Aprilov Blvd, 4002 Plovdiv, Bulgaria; yordanka.uzunova@mu-plovdiv.bg

**Keywords:** oral health, dental students, preventive care, DMFT index, Bulgaria

## Abstract

**Background:** As a fundamental component of general health, oral health is of significant global concern, with the global burden of dental diseases continuing to rise. Dentists are expected not only to provide clinical care but also to model healthy behaviours and promote oral health through education and advocacy. The knowledge, attitudes and practices of dental students—which represent critical elements of their professional development—play a pivotal role in shaping their future clinical behaviour. However, the extant literature suggests considerable variability in these domains, thus indicating that dental students do not always demonstrate the exemplary oral health practices expected of them. **Objectives:** The objective of the preliminary study was to assess the oral health practices, utilisation of preventive care, and factors influencing the oral health status of dental students at the Medical University of Plovdiv. **Methods:** A cross-sectional study was conducted among 138 first- to fifth-year dental students. The study utilised a structured, validated, adapted WHO questionnaire and a clinical examination based on the WHO Basic Oral Health Survey criteria. A comprehensive data set was collected, encompassing sociodemographic characteristics, oral hygiene behaviours, lifestyle habits, and DMFT scores. **Results:** The utilisation of preventative dental care was found to be high, with 73.2% of the student population reporting a dental visit within the previous six months. This tendency was found to be particularly pronounced among female students, who exhibited a significantly higher frequency of such visits. The prevalence of smoking was found to be significant, with 45.3% of the population reporting current smoking habits. The mean DMFT score was 3.33 (SD 3.13), predominantly driven by filled teeth. The present study found residence to be a significant factor associated with DMFT (*p* = 0.010). Specifically, rural students exhibited higher scores compared to their urban counterparts. Despite the tendency of smokers and those who brush less frequently to exhibit higher DMFT values, no statistically significant associations were identified regarding toothbrushing frequency, smoking, alcohol consumption, or dental visit frequency. **Conclusions:** The present study demonstrates that, despite the high utilisation of preventive dental care among dental students, notable disparities in oral health outcomes persist. Residence was identified as the strongest associated factor, with students from rural areas exhibiting substantially higher DMFT scores. Overall, the findings underscore the need for stronger lifestyle-focused education and targeted interventions to better prepare future oral health professionals to promote effective preventive care.

## 1. Introduction

Oral health is widely recognized as an essential component of overall well-being. The World Health Organization (WHO) defines it as a state of the mouth and facial structures that allows people to eat, speak, and function comfortably, while also supporting self-esteem and social well-being [1]. The World Dental Federation (FDI) emphasizes both the functional and psychosocial dimensions of oral health. According to Glick et al., it is a multifaceted concept reflecting an individual’s ability to speak, smile, taste, chew, and express emotions without pain, discomfort, or disease [2]. Oral health is dynamic and influenced by habits, attitudes and behaviours, as well as by evolving experiences, perceptions and environmental and social conditions throughout life [3].

Oral diseases represent a substantial global health burden, affecting an estimated 3.5 billion people worldwide [1]. It is hypothesised that the burden of these conditions may in fact exceed that of the most prevalent non-communicable diseases combined. The primary drivers of this condition are believed to be untreated dental caries, severe periodontal disease, edentulism and malignancies of the lip and oral cavity [4]. In recent decades, a general upward trend has been observed in the absolute number of oral disease cases across all age groups. In response to the evidence, the WHO has advocated for integrating oral health initiatives into national strategies for the prevention of chronic diseases. The organisation has prioritised interventions aimed at reducing tobacco and alcohol consumption, and limiting free sugars and sugar-sweetened beverages [5].

Recent epidemiological studies conducted within the Bulgarian population indicate a high prevalence and severity of periodontal diseases, which are strongly correlated with suboptimal oral hygiene practices and dietary habits. Furthermore, the prevalence of dental caries demonstrates a substantial burden across all active age cohorts, exhibiting an age-dependent prevalence pattern: 63.31% in the 45–65 age group, 52.8% in the 30–44 age group, and 36.54% among individuals aged 18–29 years [6,7]. The maintenance of optimal oral health is contingent upon consistent self-care behaviours, with particular emphasis on twice-daily toothbrushing using a fluoride-containing toothpaste. Although dental floss and mouthwash may provide additional benefits, toothbrushing remains the most effective method for preventing dental caries and gingivitis. Within the dental community, regular biannual dental check-ups are regarded as essential for the early detection and prevention of oral diseases [8,9].

In 2007, the World Health Assembly called upon member states to strengthen oral public health through the implementation of coordinated, long-term preventive policies. In response, Bulgaria initiated a national programme targeting the early stages of life, which was subsequently extended until 2025. This sustained initiative is indicative of a life-course perspective, acknowledging the significance of establishing healthy oral conditions in early life for the maintenance of oral health throughout adulthood [10,11]. Routine preventive dental examinations represent one of the elements of a comprehensive preventive framework within the domain of oral health care. It is vital that knowledge of common chronic disease risk factors is integrated into health promotion and primary prevention within dental practice. It is imperative that individuals receive consistent professional evaluation of their oral health from dentists in order to ensure that they receive comprehensive and high-quality care [12].

Dentists are expected to embody optimal health behaviours and proactively advocate for oral health among patients, colleagues, and the broader community. The scope of their function extends beyond clinical practice to encompass health education and advocacy. A plethora of studies have demonstrated that the levels of knowledge, attitudes and behaviours regarding oral health among dental students vary according to the training stage and the institution, with each domain exerting a distinct influence on the students’ oral health status [13,14,15,16,17]. The findings emphasise the necessity of enhancing the preventive orientation and health-promoting competencies within dental education to equip future professionals with the necessary skills to improve the oral health of the population.

The objective of the preliminary study was to assess the oral health practices, utilisation of preventive care, and factors influencing the oral health status of dental students at the Medical University of Plovdiv.

## 2. Materials and Methods

A pilot observational, descriptive, cross-sectional study incorporating a structured questionnaire survey was conducted between September 2024 and February 2025, among students enrolled in the Faculty of Dental Medicine at the Medical University of Plovdiv, Bulgaria. The target population comprised first- to fifth-year students. The study sample consisted of 138 students (27.8% of the eligible population). All students who met the predefined inclusion and exclusion criteria were invited to participate during the data collection period. An a priori sample size calculation was performed to ensure that the minimum required number of participants was achieved. The inclusion criteria for participation were as follows: (1) Bulgarian students; (2) students enrolled at the Medical University of Plovdiv; (3) provision of informed consent. The exclusion criteria comprised the following: (1) refusal on the part of the participant; (2) exchange or foreign students; and (3) self-reported medical conditions that could substantially influence oral health behaviours or compromise reliable participation.

In accordance with the WHO STEPwise Approach to Chronic Disease Risk Factor Surveillance [18], participants first completed a structured questionnaire and subsequently underwent a clinical dental examination. Ethical approval for the study was obtained from the Scientific Ethics Committee of the Medical University of Plovdiv, Bulgaria (Approval No. 6/4 July 2024). All procedures were conducted in accordance with the ethical standards of the institutional research committee and adhered to the principles outlined in the Declaration of Helsinki of the World Medical Association. Participation was voluntary, and informed consent was obtained from all respondents prior to data collection.

### 2.1. Study Design

A pilot observational, descriptive, cross-sectional study was conducted using a structured, self-administered questionnaire. The questionnaire was adapted from the methodological guidance of the WHO Oral Health Surveys: Basic Methods [18]. The specific items under discussion were developed by the research team in alignment with the recommended areas. The final questionnaire comprised 33 items, which were divided into three sections: (1) self-assessment of oral and dental health; (2) harmful habits related to oral health; and (3) dietary risk factors. Demographic information, including gender, age, and place of residence, was collected at the commencement of the survey. Responses were recorded on a Likert scale, with an additional ‘don’t know/cannot answer’ option, which was coded as missing. Questionnaires that exhibited a minimum of nine missing responses were excluded from the statistical analysis.

### 2.2. Clinical Examination

Following the completion of the questionnaire, each participant was subjected to a clinical dental examination by a dentist. The assessment of dental status was conducted in accordance with the standardised diagnostic criteria of the WHO, thereby ensuring a uniform and reliable evaluation process.

### 2.3. Basic Oral Health Assessment Tool

To ensure linguistic, cultural, and conceptual equivalence, the questionnaire was translated and adapted into Bulgarian following WHO methodological guidance. The process included forward translation by bilingual experts, expert panel review, and back-translation to confirm semantic consistency. Cognitive testing with 10 respondents demonstrated good clarity and comprehensibility.

The temporal stability of the questionnaire was assessed through a test–retest procedure with 10 participants. After providing feedback on clarity and comprehensibility, the questionnaire was re-administered two weeks later, yielding strong correlations between administrations (r = 0.82–0.94), indicating notable stability.

Pilot psychometric testing demonstrated satisfactory measurement properties. The instrument showed good internal consistency (Cronbach’s α = 0.78), strong test–retest reliability (r = 0.83), and high content validity (CVI = 0.89). This analysis supported the robustness of the instrument for use in oral health research [19]. Test–retest stability was further evaluated using Bland–Altman plots and the intraclass correlation coefficient (ICC) [19,20].

All participants underwent a clinical oral health assessment in accordance with the standardised methods and criteria outlined in the WHO Oral Health Surveys: Basic Methods [21]. Prior to the examination, demographic information was collected, including identification numbers, names, sex, age, and place of residence. Examiner calibration demonstrated excellent intra-examiner agreement (κ > 0.80) [22].

The oral examinations were conducted by a dentist with the relevant qualifications, using a standard dental probe, mirror, and a dental reflector light with an intensity of 20,000 lux. During the examination, the following parameters were systematically recorded: the total number of teeth present, the number of restorations (i.e., fillings), the presence of carious lesions, the number of missing teeth, and teeth restored with direct restorations (composite obturations) or indirect fixed prosthetic restorations (crowns).

### 2.4. DMFT Index

The extent and incidence of dental caries were assessed using the universally recognised Decayed, Missing, and Filled Teeth (DMFT) index, in accordance with the guidelines of the WHO [16,19]. The DMFT score for each participant was derived from the clinical examination findings, which included the number of teeth that were decayed and left untreated (DT), missing due to caries (MT), or filled as a result of caries (FT). The DMFT value for an individual was determined as the sum of DT + MT + FT. It is acknowledged that the total number of permanent human teeth is 32, and consequently, the DMFT index ranges from a minimum of 0 (no caries experience) to a maximum of 32 (all teeth affected).

### 2.5. Statistical Analysis

All statistical analyses were performed using IBM SPSS Statistics for Windows, Version 23.0 (IBM Corp., Armonk, NY, USA). The utilisation of descriptive statistics, encompassing the mean, standard deviation (SD), and frequency distribution tables, was employed to summarise demographic characteristics and oral health variables. The Chi-square test was utilised to examine the associations between categorical variables. The distribution of the DMFT index was assessed using the Shapiro–Wilk test and visual inspection of histograms and Q–Q plots. DMFT demonstrated a clearly non-normal distribution, consistent with its nature as count data. To enhance transparency, DMFT values are reported using both mean ± SD and median with interquartile range (IQR). The differences in DMFT index values across independent variables were assessed using the Mann–Whitney U test for two-group comparisons and the Kruskal–Wallis test for comparisons involving more than two groups. It is noteworthy that all statistical tests were two-tailed, with a significance level set at *p* < 0.05. Where appropriate, 95% confidence intervals (CI) were reported in order to provide precision estimates.

Due to the exploratory nature of the study and the limited sample sizes of specific subgroups, particularly those residing in rural areas, the implementation of multivariable regression modelling was not feasible. It is imperative to note that all reported associations are therefore unadjusted (bivariate), and as such, they should be interpreted with appropriate caution. The primary objective of this exploratory investigation was to discern preliminary trends to inform future, larger-scale studies rather than to establish independent predictors.

## 3. Results

The study sample consisted of 138 dental students, of whom 87 (63%) were female and 51 (37%) were male. The mean age of the participants was 21.49 years (SD = 1.77; range: 19–28 years). The majority of participants resided in urban areas (124; 89.9%), while only 14 (10.1%) reported living in rural areas (Table 1).

### 3.1. Oral Hygiene Practices

In a survey of 138 dental students, the majority of participants (124; 89.9%) reported brushing their teeth at least twice daily, indicating adherence to recommended oral hygiene practices. Conversely, 11 students (8.0%) reported brushing only once per day. Manual toothbrushes were the predominant choice (112; 81.2%), followed by electric (49; 35.5%) and sonic (25; 18.1%) devices. Adjunctive oral hygiene measures were extensively implemented. Female students demonstrated a significantly higher utilisation of dental floss and mouthwash (*p* < 0.05). Fluoride-containing products, a well-established cornerstone in caries prevention, were utilised by 91 participants (65.5%). Interdental brushes (35; 25.4%) and dental irrigators (27; 19.6%) were adopted less frequently [1,23].

### 3.2. Dental Visits

The utilisation of preventative dental care was prevalent among the study participants. The majority of respondents (101; 73.2%) had visited a dentist within the previous six months, while 31 (22.5%) reported a visit within the past year. A mere 6 participants (4.3%) reported a dental visit that occurred more than a year prior. The predominant reason for the most recent dental visit was a routine check-up (83; 60.1%), followed by consultation (22; 15.9%) and dental treatment (20; 14.6%). In the present study, pain was reported as a reason by 10 participants (7.2%), while 3 (2.2%) participants could not recall the reason. A statistically significant difference was found between the sexes in terms of the frequency of dental visits, with female students reporting a higher frequency of such visits than their male counterparts (*p* < 0.05).

### 3.3. Lifestyle Habits

A total of 63 students (45.3%) reported current cigarette smoking, highlighting a notably high prevalence of tobacco use within this cohort. Alcohol consumption was reported by 23 participants (16.6%), with 58 (41.7%) engaging in physical activity at least three times weekly.

### 3.4. Self-Assessment of Dental Status

The self-perception of dental health was, in general, positive. The majority of subjects evaluated their dental condition as either “very good” (68 subjects, 49.3%) or “excellent” (26 subjects, 18.8%). A further 33 (23.9%) described their dental status as good. A mere minority reported less favourable assessments, with average (1; 0.7%) and fair (10; 7.2%) being the most common. It is noteworthy that none of the subjects evaluated their dental condition as being poor. As demonstrated in Table 2, a comprehensive overview of oral hygiene practices, tobacco use, and alcohol consumption is provided among male and female dental students.

With regard to oral hygiene, a significantly higher percentage of females (60.9%) reported cleaning their teeth twice or more daily compared to males (29.0%). This discrepancy was found to be statistically significant (*p* = 0.006). The analysis revealed no significant differences in tobacco use between males and females (*p* = 0.973). The distribution of tobacco use across daily, occasional, and non-use categories was comparable for both genders. However, a significant difference was revealed by alcohol use (*p* = 0.016): daily consumption was reported only among males (2.2%), while females were more likely to consume alcohol occasionally, with 33.3% reporting use two to three times per month and 23.2% reporting sometimes or never. These findings underscore the existence of sex-related disparities in oral hygiene and alcohol consumption habits, while tobacco use appeared to be equally distributed across both groups. As illustrated in Table 3, significant disparities in dietary habits were observed between the two genders.

The ingestion of soft drinks devoid of added sugar was found to be without significant difference (*p* > 0.05), although females reported a higher weekly intake. In contrast, consumption of carbonated soft drinks with added sugar differed significantly (*p* = 0.001), with males more likely to consume them daily (8.0%) and females more likely to rarely or never consume them (29.1%). Sugared coffee intake was found to be significantly higher among females (15.9% vs. 6.5%, *p* = 0.025), while juice consumption was also found to be greater among females, with higher weekly and monthly intake. This difference was found to be statistically significant (*p* = 0.047).

The investigation revealed that there was no statistically significant difference between the sexes with regard to physical activity (*p* = 0.064). The majority of females (26.1%) engaged in exercise two to three times per week, while the male population exhibited a more even distribution across daily and weekly activity patterns.

As illustrated in Table 4, the distribution of the DT, MT, FT and DMFT indices among the dental students (N = 138) is demonstrated across demographic and behavioural variables.

The mean DMFT index among the dental students was 3.33 (SD 3.13), with the majority of the score attributable to filled teeth (FT = 3.04), while decayed teeth (DT = 0.25) and missing teeth (MT = 0.04) contributed minimally. The DMFT index exhibited a non-normal distribution (Shapiro–Wilk *p* < 0.001). The median DMFT was 3.00, with an interquartile range (IQR) of 0.00–5.00, indicating substantial variability in caries experience within the sample. Place of residence emerged as the only significant factor associated with DMFT (*p* = 0.024). Students residing in rural areas had substantially higher DMFT values (5.36, SD 3.713) than their urban counterparts (3.10, SD 2.986), indicating that environmental and socioeconomic factors may influence caries experience. This finding directly supports the study aim by identifying a demographic factor with measurable impact on oral health status.

Behavioural variables were also examined as part of the study aim. Smokers, students brushing less frequently, and those who did not consume alcohol showed slightly higher DMFT values, but none of these differences reached statistical significance. Dental-visit frequency was likewise unrelated to DMFT outcomes. Although non-significant, these behavioural patterns provide contextual insight and may warrant further investigation in larger samples.

**Figure 1:**
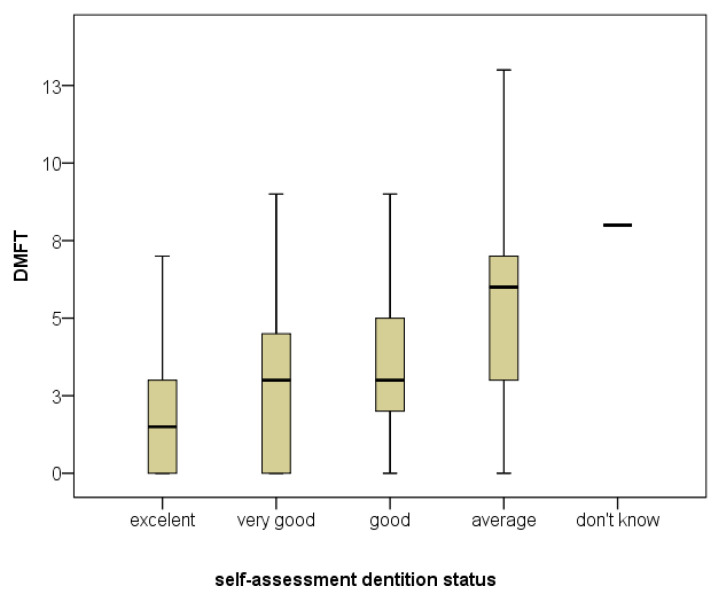
Distribution of DMFT Index by Self-Assessed Dentition Status: Box-Plot Analysis (Kruskal–Wallis Test, *p* = 0.003).

Figure 1 illustrates the relationship between self-assessed dental status and DMFT. Students rating their oral health as excellent had the lowest DMFT scores, while those reporting good or fair status showed progressively higher values. This relationship supports the study aim by linking subjective perception with objective oral health status.

The figure under consideration displays a clear gradient: higher positive self-assessments are associated with lower DMFT scores, while lower positive self-assessments are associated with higher DMFT scores.

## 4. Discussion

This study examined oral hygiene practices, lifestyle habits, and caries experience among dental students to identify strengths and areas requiring improvement.

Most students reported brushing twice daily and using fluoride toothpaste, a finding that aligns with prior research indicating superior oral hygiene among dental students. As Kassim et al. emphasised, the use of fluoridated toothpaste is of paramount importance in the control of dental caries among students [24]. Previous studies have similarly shown that dental students and young adults demonstrate better oral hygiene behaviours than non-dental peers, largely due to their professional training and increased awareness [25]. However, the low use of interdental brushes (25.4%) and dental irrigators (19.6%) suggests gaps in comprehensive plaque-control strategies. A significant gender disparity was observed, with female students more frequently using dental floss and mouthwash, consistent with literature reporting superior oral hygiene practices among women [26].

The findings demonstrate a high level of utilisation of preventive dental care among dental students, consistent with studies from other countries reporting favourable oral health-related knowledge, attitudes, and behaviours in this population [17]. Evidence suggests that the structure of dental education—particularly peer-to-peer clinical training—plays a pivotal role in shaping students’ preventive behaviours, as regular engagement with evidence-based practices reinforces both adherence to and promotion of optimal oral health behaviours [27].

Despite their professional training, 45.3% of students reported current cigarette smoking, a prevalence that exceeds levels typically observed among health professionals and underscores a concerning pattern identified in our study. Comparable findings have been reported in Poland and Italy (21–28%) and in Iraq (37%) [28,29]. In a similar vein, Thomas et al. documented rates of approximately 30% among dental students in India, and Kaiss et al. observed comparable figures in Morocco [30,31]. A broader review indicates that smoking prevalence among dental students ranges from 11% to 42%, depending on geographic and cultural context [32,33]. This finding indicates that tobacco use remains a persistent behavioural challenge even among future oral health practitioners, revealing a clear gap between professional knowledge and personal behaviour and underscoring the need for stronger educational and preventive strategies within dental curricula.

In our study, self-perceived dental health was generally positive, aligning with the low levels of untreated decay observed (DT = 0.25). The mean DMFT index of 3.33 recorded in our sample was substantially lower than values typically reported in general adult and adolescent populations. In Bulgaria, Nenov et al. reported a mean DMFT of 15.7 in the active-age population [6], while Olczak-Kowalczyk et al. found a mean DMFT of 6.50 among Polish adolescents [34]. Globally, Peres et al. emphasised that dental caries remains the most prevalent disease worldwide, with adult DMFT values often exceeding 10 [35]. The comparatively low DMFT in the present cohort may reflect early exposure to preventive programmes, including fluoride varnish, supervised toothbrushing, and sealant application, which have been shown to reduce caries incidence [36,37].

Residence emerged as the only factor significantly associated with DMFT, with rural students exhibiting higher values (5.36 vs. 3.10). This finding aligns with evidence that rural populations face systemic barriers to oral health care, including reduced access to services, lower utilisation of preventive resources, and limited oral health literacy [38]. In a similar manner, Sidhu et al. emphasised that rural dental health disparities in South Asia exhibited a robust correlation with diminished access and utilisation of preventive services [39].

Although smoking, brushing frequency, and alcohol consumption showed trends toward higher DMFT, these associations were not statistically significant. Similar non-significant patterns have been reported in Turkish dental student cohorts, where lifestyle factors influenced DMFT but were moderated by educational exposure [40]. The lack of significance in the present study may be attributable to the limited sample size.

The present study is subject to several limitations. The reduced sample size may have constrained the statistical power to detect meaningful associations. It is acknowledged that self-reported behaviours, including smoking and tooth-brushing frequency, may be subject to recall bias or social desirability bias. The cross-sectional design of the study further restricts the ability to draw causal inferences, and unmeasured factors—such as socioeconomic status, detailed dietary patterns, and previous dental interventions—may have introduced residual confounding. Furthermore, the sample was drawn from a single institutional population, which may limit the generalisability of the findings.

A key methodological limitation is the reliance on unadjusted bivariate analyses. While residence was identified as the sole statistically significant factor associated with DMFT, the absence of multivariable modelling precludes conclusions regarding the independent effect of this variable relative to others. The reduced number of participants from rural areas has a detrimental effect on the stability and precision of subgroup estimates. It is recommended that future studies with larger and more balanced samples incorporate adjusted regression analyses in order to validate and expand upon these preliminary observations.

## 5. Conclusions

The present study demonstrates that, despite the high utilisation of preventive dental care among dental students, differences in oral health outcomes remain evident. In this pilot sample, residence was the only variable to demonstrate a statistically significant unadjusted association with DMFT scores. The high prevalence of smoking among future oral health professionals further highlights an important inconsistency, suggesting engagement in behaviours that may compromise their own oral health. Although no significant associations were found between toothbrushing frequency or alcohol consumption and oral health outcomes, the findings indicate that factors beyond individual behaviours may also contribute. The preliminary results suggest that place of residence may influence oral health outcomes, a topic that warrants further investigation through multivariable modelling in future studies with larger and more representative samples. These observations emphasise the necessity of enhancing the preventive orientation of dental education, particularly with respect to modifiable lifestyle risk factors and the provision of personalised support for students from diverse residential backgrounds. It is imperative that these components are enhanced to ensure that future oral health professionals are adequately prepared to promote effective preventive practices and contribute to improving population oral health.

## Figures and Tables

**Table 1:** The demographic, behavioural and oral health characteristics of a sample of dental students (N = 138).

Characteristics	N	%
Sex		
Male	51	37
Female	87	63
Place of residence		
Urban area	124	89.9
Rural area	14	10.1
Toothbrush type (Multiple answers allowed)		
Manual/classic	112	81.2
Electric	49	35.5
Sonic	25	18.1
Tooth brushing frequency (≥2 times/day)	124	89.9
Oral hygiene practices (Multiple answers allowed)		
Fluoride toothpaste	91	65.9
Chlorhexidine toothpaste	18	13.0
Dental floss	92	66.7
Mouthwash	83	60.1
Intradental brushes	35	25.4
Dental irrigator	27	19.6
Dental visit		
Less than 6 months	101	73.2
6 months–1 year	31	22.5
1 year–2 years	2	1.4
More than 2 years	4	2.9
Reason for the most recent dental visit		
consultation	22	15.9
pain (dental, oral, facial, etc.)	10	7.2
dental treatment	20	14.6
regular dental visits	83	60.1
don’t remember	3	2.2
Lifestyle habits		
Current cigarette smoking (yes)	63	45.3
Frequency of alcohol use (>3 times weekly)	23	16.6
Frequency of physical activity (>3 times weekly)	58	41.7
Self-assessed of dental status		
excellent	26	18.8
very good	68	49.3
good	33	23.9
average	1	0.7
fair	10	7.2
poor	-	-
	Mean	SD
Age, (range 19–28) years	21.49	1.77

Descriptive statistics (frequencies, percentages, means and standard deviations).

**Table 2:** Gender differences in oral hygiene practices and lifestyle behaviours among the study participants (N = 138).

Frequency of	Male Students (n = 51)	Female Students (n = 87)	*p* Value *
Tooth cleaning			
twice or more times a day	40 (29.0)	84 (60.9)	0.006 *
once a day	9 (6.5)	2 (1.4)	
several times a week	2 (1.4)	1 (0.7)	
Tobacco use			
every day	15 (10.9)	23 (16.7)	0.973
several times a week	3 (2.2)	5 (3.6)	
once a week	2 (1.4)	3 (2.2)	
several times a month	1 (0.7)	3 (2.2)	
sometimes	2 (1.4)	6 (4.3)	
never	28 (20.3)	47 (34.1)	
Alcohol use			
every day	3 (2.2)	-	0.016 *
2–3 times a week	11 (8.0)	9 (6.5)	
2–3 times a month	26 (18.8)	46 (33.3)	
sometimes/never	11 (8.0)	32 (23.2)	

Pearson Chi-Square test, * *p* < 0.05.

**Table 3:** Gender differences in beverage consumption and physical activity (N = 138).

Frequency of Consumption	Male Students N(%)	Female Students N(%)	*p* Value *
Sugar-free soft drinks			
every day	6 (4.3)	3 (2.2)	0.087
several times a week	7 (5.1)	23 (16.7)	
once a week	10 (7.2)	9 (6.5)	
several times a month	12 (8.7)	16 (11.6)	
rarely/never	16 (11.6)	36 (26.1)	
Sugar-sweetened carbonated drinks			
every day	11 (8.0)	3 (2.2)	0.001 *
several times a week	9 (6.5)	22 (15.9)	
once a week	6 (4.3)	5 (3.6)	
several times a month	14 (10.1)	17 (12.3)	
rarely/never	11 (8.0)	40 (29.1)	
Sugar-sweetened coffee			
every day	9 (6.5)	22 (15.9)	0.025 *
several times a week	8 (5.8)	12 (8.7)	
once a week	4 (2.9)	1 (0.7)	
several times a month	12 (8.7)	7 (5.1)	
rarely/never	18 (13.1)	45 (32.6)	
Fruit juices			
every day	2 (1.4)	2 (1.4)	0.047 *
several times a week	6 (4.3)	15 (10.9)	
once a week	9 (6.5)	16 (11.6)	
several times a month	9 (6.5)	26 (18.8)	
rarely/never	25 (18.3)	28 (20.3)	
Frequency of physical activity			
every day	14 (10.1)	19 (13.9)	0.064
4–6 times a week	14 (10.1)	11 (8.0)	
2–3 times a week	10 (7.2)	36 (26.1)	
once a week	5 (3.6)	4 (2.9)	
several times a month	8 (5.8)	16 (11.6)	
rarely/never	-	1 (0.7)	

Pearson Chi-Square test, * *p* < 0.05.

**Table 4:** Oral health status (DT, MT, FT, DMFT) among participants across sociodemographic and behavioural characteristics.

Variable	Healthy N (%)	DT		MT		FT		DMFT Index		*p* Value
		MEAN	SD	MEAN	SD	MEAN	SD	MEAN	SD	
Sex										
Male	42 (30.4)	0.25	0.627	0.12	0.516	2.88	2.855	3.18	3.146	0.296
Female	75 (54.3)	0.24	0.698	-	-	3.26	2.822	3.51	3.121	
Age										
19–23	109 (79)	0.22	0.655	0.05	0.333	2.94	2.755	3.24	3.052	0.239
24–29	8 (5.8)	0.54	0.766	-	-	4.50	3.536	4.54	3.865	
Place of residence										
Urban	107 (77.5)	0.19	0.536	0.03	0.283	2.81	2.814	3.10	2.986	0.024 *
Rural	10 (7.2)	0.71	1.326	0.14	0.535	4.50	2.739	5.36	3.713	
Smoking behaviour										
no	69 (50)	0.12	0.273	0.01	0.115	2.84	2.664	2.97	2.773	0.185
yes	48 (34.8)	0.40	0.429	0.08	0.451	3.27	3.459	3.75	3.598	
Alcohol consumption										
no	43 (31.2)	0.35	0.897	0.06	0.381	3.44	2.746	3.79	3.174	0.207
yes	95 (68.8)	0.20	0.538	-	-	2.85	2.877	3.12	3.101	
Tooth-brushing behaviour										
two or more times	124 (89.9)	0.14	0.363	-	-	2.64	2.790	2.79	3.017	0.513
less two times	14 (10.1)	0.26	0.697	0.05	0.334	3.08	2.853	3.39	3.146	
Since last dental visit										
<6 months	101 (73.2)	0.24	0.637	0.04	0.314	3.11	2.942	3.36	3.214	0.996
>6 months	37 (26.8)	0.35	0.753	0.05	0.329	2.84	2.566	3.24	2.919	

“Healthy” = DT = 0 and MT = 0; Decayed Teeth (DT), Missing Teeth (MT), Filled Teeth (FT); *p*-value is related to difference subgroups for DMFT, Mann–Whitney U test, * *p* < 0.05.

## Data Availability

The original contributions presented in this study are included in the article. Further inquiries can be directed to the corresponding author.
